# High-Flux Hemodialysis and High-Volume Hemodiafiltration Improve Serum Calcification Propensity

**DOI:** 10.1371/journal.pone.0151508

**Published:** 2016-04-11

**Authors:** Marijke Dekker, Andreas Pasch, Frank van der Sande, Constantijn Konings, Matthias Bachtler, Mauro Dionisi, Matthias Meier, Jeroen Kooman, Bernard Canaud

**Affiliations:** 1 Department of Internal Medicine, Division of Nephrology, Maastricht University Medical Center, Maastricht, The Netherlands; 2 Department of Internal Medicine, Division of Nephrology, Catharina Hospital Eindhoven, Eindhoven, The Netherlands; 3 Department of Clinical Chemistry, University Hospital Bern, Bern, Switzerland; 4 Department of Clinical Research, University of Bern, Bern, Switzerland; 5 Calciscon AG, Bern, Switzerland; 6 Fresenius Medical Care, Bad Homburg, Germany; Medical University of Graz, AUSTRIA

## Abstract

**Background:**

Calciprotein particles (CPPs) may play an important role in the calcification process. The calcification propensity of serum (T_50_) is highly predictive of all-cause mortality in chronic kidney disease patients. Whether T_50_ is therapeutically improvable, by high-flux hemodialysis (HD) or hemodiafiltration (HDF), has not been studied yet.

**Methods:**

We designed a cross-sectional single center study, and included stable prevalent in-center dialysis patients on HD or HDF. Patients were divided into two groups based on dialysis modality, were on a thrice-weekly schedule, had a dialysis vintage of > 3 months and vascular access providing a blood flow rate > 300 ml/min. Calcification propensity of serum was measured by the time of transformation from primary to secondary CPP (T_50_ test), by time-resolved nephelometry.

**Results:**

We included 64 patients, mean convective volume was 21.7L (SD 3.3L). In the pooled analysis, T_50_ levels increased in both the HD and HDF group with pre- and post-dialysis (mean (SD)) of 244(64) - 301(57) and 253(55) - 304(61) min respectively (P = 0.43(HD vs. HDF)). The mean increase in T_50_ was 26.29% for HD and 21.97% for HDF patients (P = 0.61 (HD vs. HDF)). The delta values (Δ) of calcium, phosphate and serum albumin were equal in both groups. Baseline T_50_ was negatively correlated with phosphate, and positively correlated with serum magnesium and fetuin-A. The ΔT_50_ was mostly influenced by Δ phosphate (*r* = -0.342; P = 0.002 HD and *r* = -0.396; P<0.001 HDF) in both groups.

**Conclusions:**

HD and HDF patients present with same baseline T50 calcification propensity values pre-dialysis. Calcification propensity is significantly improved during both HD and HDF sessions without significant differences between both modalities.

## Introduction

In dialysis patients vascular calcifications are an independent predictor for cardiovascular and all-cause mortality [1]. Vascular calcification is also a risk factor for increased arterial stiffness, which is also an independent risk factor for mortality in this population [2]. An initiating factor for this increased vascular calcification is hyperphosphatemia [3–5], due to impaired phosphate (P) removal by dialysis [6]. In serum, P may form calciprotein particles (CPPs) with calcium (Ca) and fetuin-A [7]. CPPs store P and Ca in a non-crystalline soluble form which allows their removal from the circulation [8]. The levels of circulating CPPs have been related to vascular stiffness and have been reported to possess pro-inflammatory properties in vitro [3, 9, 10]. The formation of CPPs is a two-step process. First Ca, P and fetuin-A bind together and form an amorphous colloidal calcium-phosphate nanoparticle [11], called primary CPP [7, 12, 13]. Secondly, they transform into topologically stable elongate spindle-shaped structures containing proteins and hydroxy-apatite, called secondary CPP [14, 15]. The transition time (T_50_) between primary and secondary CPP is thought to reflect the intrinsic inhibitory capacity of the serum to prevent Ca and P from precipitating [13]. T_50_ is accelerated by high serum concentrations of Ca and P and delayed by high magnesium concentrations [12, 13]. Fetuin-A is an indispensable protein in CPP formation [9, 12, 16].

Recently, a novel in vitro blood test was developed which measures the transformation time point from primary to secondary CPP [13]. This test, which is a functional composite of established non-traditional risk factors, has already been demonstrated to be highly predictive of all-cause mortality in stage 3 and 4 chronic kidney disease (CKD) and to outperform its individual components in this regard, whereas also a relation between lower T_50_ values and an increase in arterial stiffness was observed. [12].

Compared to conventional high-flux hemodialysis (HD), hemodiafiltration (HDF) leads to improved removal of larger molecular weight uremic toxins and a reduction in inflammatory markers [17]. A recent randomised trial showed a beneficial effect of HDF on all-cause and cardiovascular mortality [18]. Moreover, a beneficial effect of HDF on vascular stiffness was suggested [19], although this was not observed in other studies [20, 21]. The effect of HD or HDF on calcification propensity has not been studied yet. Although CPPs are much larger than the pore sizes of the dialysis membrane and a direct effect of HD or HDF on their removal is therefore unlikely, several studies have showed an improved P removal in HDF patients compared to HD patients [22, 23]. This, however is not a uniform finding, and may depend on membrane characteristics and other treatment characteristics [24]. Moreover, HDF could also increase the removal of factors involved in vascular calcification, such as FGF-23 (mw 32 kDa) and sclerostin (mw 23 kDa) [25–27]. On the other hand, at least theoretically, fetuin-A (mw 59 kDa) could also to some degree be removed by HDF. Whether the effects of HDF also translate into a difference in calcification propensity of serum, as measured by the level of T_50_ has not yet been estimated. This cross-sectional study was conducted to compare the calcification propensity as determined by the T_50_-test in patients treated with HD and HDF in order to shed more light on potential mechanisms explaining the reported differences in cardiovascular outcomes between HD and HDF.

## Methods

### Study population

For this cross-sectional single center study we included adult, chronic HD and HDF patients treated in the Catharina Hospital Eindhoven, The Netherlands. We included all stable dialysis patients of our clinic on a trice weekly 4-hour schedule, with a dialysis vintage of minimum 3 months, an arterio-venous fistula enabling double needle vascular access with a blood flow rate of at least 300ml/min. Exclusion criteria were patients with active illnesses and hospitalized patients. In total 64 patients gave written informed consent for participation in this study. Blood samples were drawn during all in centre dialysis treatments in the first week of October 2014, to analyse the possible effect of differences in pre-dialysis values after different in time intervals between two dialysis sessions. This study was approved by the Medical Board from the Catharina Hospital and conducted by good clinical practice guidelines based on the Declaration of Helsinki.

### Biochemical measurements

Sera from both the HD and HDF patients were drawn before and after dialysis during all in centre dialysis sessions in one week. Sera were analysed for Ca, magnesium, albumin, P, bicarbonate and CRP on a routine automated analyser (Cobas c502 immunochemistry analyser (Roche Diagnostics, Almere, The Netherlands). For the analysis of fetuin-A and the CPP transformation time (T_50_) an additional 5ml, non-additive BD-vacutainer glass serum tube was collected. Within 240 minutes after collection these samples were centrifuged at room temperature (20°C) for 15 minutes. Of this 800 ul of serum was stored at 4°C and send to the lab of Calciscon AG in Bern, Switzerland, where they were analysed within 72 hours after collection.

The serum fetuin-A concentration was measured by nephelometry, a test first established by the Jahnen-Dechent group [28]. T_50_ was determined by the method described by Pasch et al. [13]. Samples were supersaturated by adding Ca (10mM) and P (6mM) to initiate the formation of primary CPP. The time of spontaneous transformation to secondary CPPs was measured by a nephelostar nephelometer (BMG Labtech, Ortenberg, Germany).

Correction for hemoconcentration for fetuin-A was performed by the method of Bergstrom et al. [29]: uncorrected post dialysis fetuin-A (g/L)/(1+ (Δ body weight (kg)/0.2*post dialysis body weight (kg)).

### Statistical analysis

After pooling all the pre-dialysis and post-dialysis analyses separately, off all analysis during the week, continuous variables are reported as mean and standard deviation (SD), or median and 25^th^-75^th^ percentile depending on their distributions assessed by the Kolmogorov-Smirnov test. Paired and unpaired sample T tests analyses were performed to analyse statistically significant differences between the two patients groups. For not normally distributed parameters the Mann-Withney U test was used. For associations between the different laboratory analyses we used a bivariate correlation analysis. P-values <0.05 were considered significant. The change in transformation time (T_50_) after dialysis was the primary outcome parameter in this study. In the lowest tertile of the population of Smith et al. [12] a T_50_ of 227 ±44 minutes was found. In a power analysis, with a power of 0.8 and an alpha level of 0.05, a sample size of 16 patients in each group would be needed to show a difference of 20% between both groups. However, as the differences between HD and HDF might be smaller than the assumed 20% we chose to include 30 patients in each group. Analyses were performed with SPSS statistics version 19.0 (IBM Corporation, Chicago, IL, USA).

## Results

### Study population

A total of 64 patients participated in this study, 30 patients on HD and 34 patients on HDF. The mean convection volume during one HDF session was 21.7L (3.3L). All patients used the non-calcium containing phosphate binder Sevelamer (Renvela^®^). With exception of a lower pre-dialysis Ca (2.31 vs. 2.24 mmol/L, P = 0.01) and a higher magnesium (1.88 vs. 1.80 mmol/L, P = 0.04) in the HD group, a higher percentage of men and a higher target weight in the HDF group, there were no statistically significant differences between the baseline values of the two groups (Table 1, Tables 2 and 3).

**Table 1:** Patients and dialysis characteristics, presented as mean (standard deviation) or median (25^th^-75^th^ interquartile range).

	High-flux hemodialysis (n = 30)		Hemodiafiltration (n = 34)		p-value
Age (years)	71	69–81	69	61–78	0.80
Male Gender	38.2%		61.8%		0.22
Target weight (kg)	70.7	16.2	79.9	13.6	0.02
Convection volume (L)			21.7	3.3	
Kt/V per week	4.7	1.0	4.8	1.2	0.59
Access flow (ml/min)	1280	655.2	1233	456.3	0.74
Dialysis vintage (months)	51	24–86	49	19–70	0.80
Dialysate sodium (mmol/L)	137.4	0.89	137.4	0.90	0.83
Dialysate potassium (mmol/L)	2.12	0.35	2.12	0.32	0.71
Dialysate bicarbonate (mmol/L)	34.83	1.46	34.82	1.17	0.23
Serum pre-dialysis bicarbonate (mmol/L)	23.5	2.0	23.6	2.4	0.25
CRP (mg/L)	9.6	15.6	9.6	15.0	0.99

Footnote: The dialysate concentrations of calcium (1.50 mmol/L), magnesium (1.0 mmol/L), acetate (3.0 mmol/l), glucose (0.991g/L) and chloride (106 mmol/L), were equal in all patients.

**Table 2:** Pooled pre- and post-dialysis laboratory values of high-flux hemodialysis patients.

	Pre-dialysis		Post-dialysis			Difference between pre- and post-dialysis		
	Mean	SD	mean	SD	p	Percentage	25^th^ and 75^th^ percentile	
Albumin (g/L)	38.53	4.70	43.26	5.77	<0.001	13.15	3.63	22.89
Calcium (mmol/L)*	2.24	0.13	2.46	0.09	<0.001	10.20	6.14	12.45
Phosphate (mmol/L)	1.39	0.37	0.70	0.14	<0.001	-47.91	-56.11	-43.24
Magnesium (mmol/L)	0.94	0.17	0.81	0.06	<0.001	-12.57	-21.05	-5.06
Fetuin-A (g/L)	0.38	0.07	0.41	0.08	<0.001	9.55	0.00	19.35
T_50_ (min)	244	64	301	57	<0.001	26.29	12.12	35.81

*Calcium corrected for serum albumin concentration (g/L).

**Table 3:** Pooled pre- and post-dialysis laboratory values of high-volume hemodiafiltration patients.

	Pre-dialysis		Post-dialysis			Difference between pre- and post-dialysis		
	mean	SD	mean	SD	p	Percentage	25^th^ and 75^th^ percentile	
Albumin (g/L)	37.62	4.64	41.44	5.53	<0.001	11.03	0.29	19.60
Calcium (mmol/L)*	2.31	0.18	2.47	0.12	<0.001	7.83	4.11	12.26
Phosphate (mmol/L)	1.37	0.41	0.67	0.20	<0.001	-47.49	-60.66	-39.73
Magnesium (mmol/L)	0.90	0.17	0.79	0.07	<0.001	-9.97	-18.75	-2.56
Fetuin-A (g/L)	0.40	0.07	0.41	0.08	<0.001	5.25	-2.5	12.5
T_50_ (min)	253	55	304	61	<0.001	21.97	11.41	34.18

*Calcium corrected for serum albumin concentration (g/L).

In the dialysis fluid used in this cohort, values of calcium (1.5 mmol/L), magnesium (1.0 mmol/L), acetate (3.0 mmol/l), glucose (0.99 g/L) and chloride (106 mmol/L), were equal in all patients. Dialysate concentrations of sodium, potassium and bicarbonate were individually adjusted, no differences were found between the two patients groups (Table 1). In all patients the low molecular weight heparin (dalteparin) was used to prevent coagulation during the dialysis session, dosage was based on pre-dialysis weight below 50kg 2500IE and above 50kg 5000IE, for both HD and HDF patients.

Blood samples were taken on all three dialysis sessions during one week. For the main analysis pre-dialysis and post-dialysis samples of all dialysis sessions during one week were pooled. In total, we analysed 87 samples pre- and post-dialysis in the HD group and 101 samples pre- and post-dialysis in the HDF group. In addition, the pre-dialysis T_50_ values were compared between the longest and shorter dialysis intervals.

### Influence of dialysis on calcification propensity and fetuin-A levels

**Fig 1:**
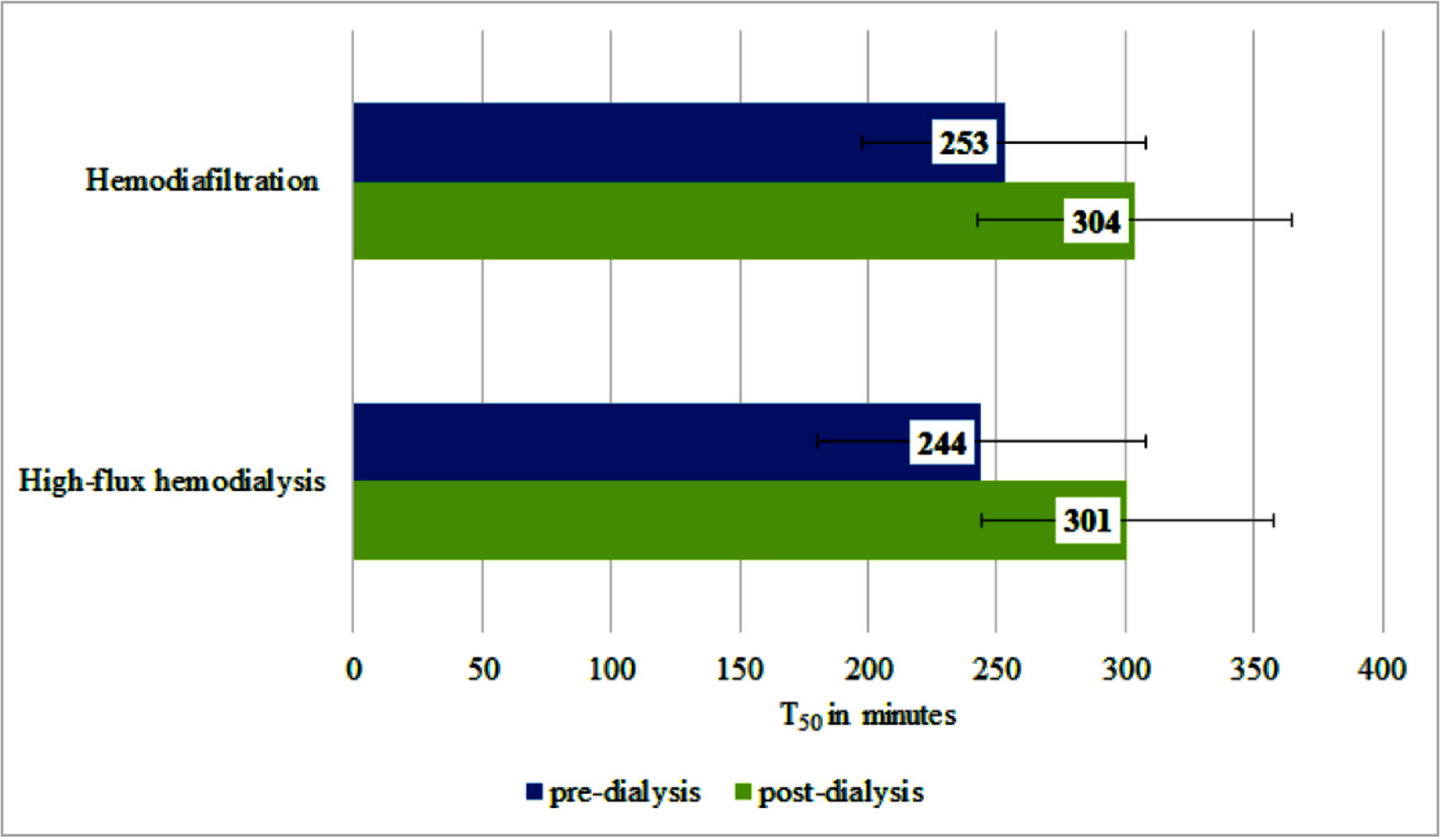
Effects of hemodialysis and hemodiafiltration on calcification propensity (T_50_), presented as mean and standard deviation.

T_50_ significantly increased (i.e. improved) after the dialysis session in both the HD and HDF group, delta T_50_ were 56 minutes (95% CI 47.31–63.95) and 51 minutes (95% CI 42.68–60.11) respectively, without significant differences between the HD and HDF groups (Fig 1). The percentage of increase of T_50_ after dialysis was 26.29% for the HD and 21.97% for the HDF group (Tables 2 and 3).

The fetuin-A concentration was influenced by both the HD and HDF session, in a minimal way with a delta value of 0.03 and 0.02 for HD and HDF respectively. But when corrected for hemoconcentration the fetuin-A concentration was not significantly influenced by HD (mean Δfetuin-A 0.003 (95% CI -0.01–0.12; P = 0.51)), but was by HDF (mean Δfetuin-A 0.013 (95% CI 0.003–0.23; P = 0.01)). Although the decline in plasma levels was small, HDF had a significantly larger effect on the change in fetuin-A concentrations as compared to HD (P = 0.002). After correction for hemoconcentration, the change of fetuin-A concentrations in the HD group was -0.46% and -3.39% for the HDF group, this could suggest some removal of fetuin-A during HDF (Table 4).

**Table 4:** Fetuin-A post-dialysis values corrected for hemoconcentration based on ΔBW [29]. Data presented as mean ± SD, mean delta between pre- and post-dialysis values. Percentage of change, with 25^th^ and 75^th^ percentile.

	Pre-dialysis		Post-dialysis		Difference between pre- and post-dialysis						
	mean	SD	mean	SD	mean Δ	95% CI		p	%	25^th^ -75^th^	
HD fetuin-A (g/L)	0.37	0.06	0.36	0.07	0.003	-0.006	0.12	0.51	-0.46	-5.61	6.01
HDF fetuin-A (g/L)	**0.39**	**0.07**	**0.37**	**0.08**	**0.013**	**0.003**	**0.02**	0.01^*^	**-3.39**	**-10.59**	**3.94**

During the week, pre-dialysis P and magnesium levels significantly decreased in both groups and T_50_ levels significantly increased (i.e. improve) from 236 (±61) to 269 (±66) (P = 0.001) minutes in the HD group and from 248 (±59) to 266 (±50) (P = 0.03) minutes in the HDF group between the first and last dialysis session within one week (Tables 5 and 6).

**Table 5:** Laboratory values pre-dialysis for the separate high-flux hemodialysis sessions during the week.

	High-flux hemodialysis (n = 30)							
	First		Second		Third			
	Mean	SD	Mean	SD	Mean	SD	p^#^	p^^^
Albumin (g/L)	38.56	5.26	38.82	4.49	38.18	4.45	0.76	0.88
Calcium (mmol/L)*	2.24	0.14	2.24	0.15	2.25	0.07	0.52	1.00
Phosphate (mmol/L)	1.49	0.42	1.44	0.32	1.24	0.27	0.001	0.02
Magnesium (mmol/L)	0.97	0.16	0.95	0.11	0.91	0.12	<0.001	0.09
Fetuin-A (g/L)	0.37	0.07	0.39	0.07	0.37	0.07	0.93	0.65
T_50_ (min)	236	61	227	59	269	66	0.001	0.04

*Calcium corrected for serum albumin concentration (g/L).

^#^p-value of a paired samples T-test between first and third dialysis of the week.

^^^p-value of an ANOVA for group differences.

**Table 6:** Laboratory values pre-dialysis for the separate high-volume hemodiafiltration sessions during the week.

	High-volume hemodiafiltration (n = 34)							
	First		Second		Third			
	Mean	SD	Mean	SD	Mean	SD	p^#^	p^
Albumin (g/L)	38.09	4.11	37.34	4.89	37.51	4.93	0.35	0.79
Calcium (mmol/L)*	2.32	0.15	2.32	0.21	2.28	0.18	0.12	0.64
Phosphate (mmol/L)	1.48	0.44	1.36	0.41	1.22	0.37	<0.001	0.05
Magnesium (mmol/L)	0.93	0.20	0.89	0.16	0.86	0.16	<0.001	0.22
Fetuin-A (g/L)	0.39	0.07	0.40	0.07	0.40	0.07	0.01	0.69
T_50_ (min)	248	59	244	55	266	50	0.03	0.22

*Calcium corrected for serum albumin concentration (g/L).

^#^p-value of a paired samples T-test between first and third dialysis of the week.

^p-value of an ANOVA for group differences.

### Biochemical parameters

**Fig 2:**
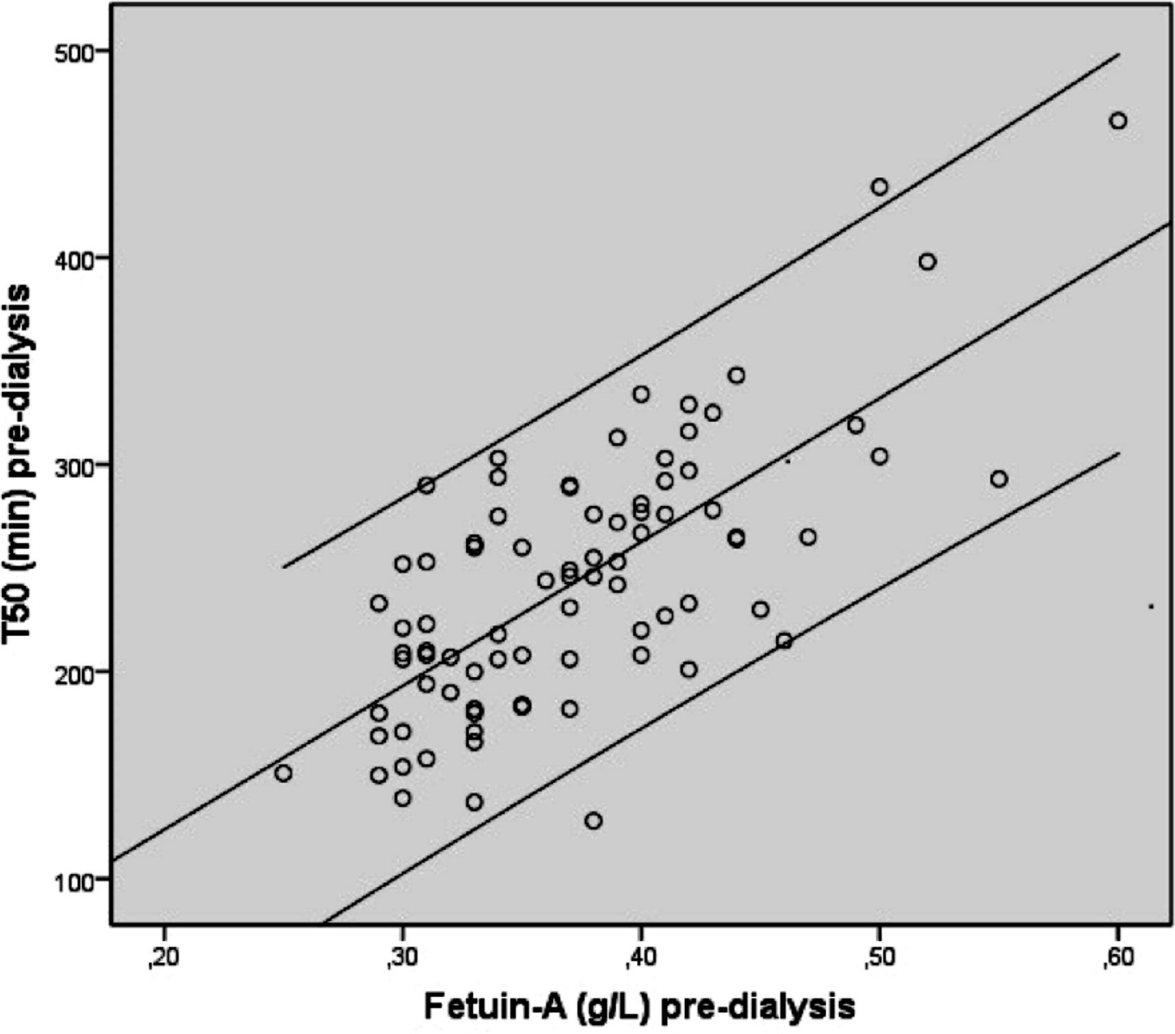
Bivariate correlation of pooled pre-dialysis T_50_ (minutes) and fetuin-A (g/L) analysis in hemodialysis patients. Fig 2 (legend): *r* 0.713 P<0.001.

**Fig 3:**
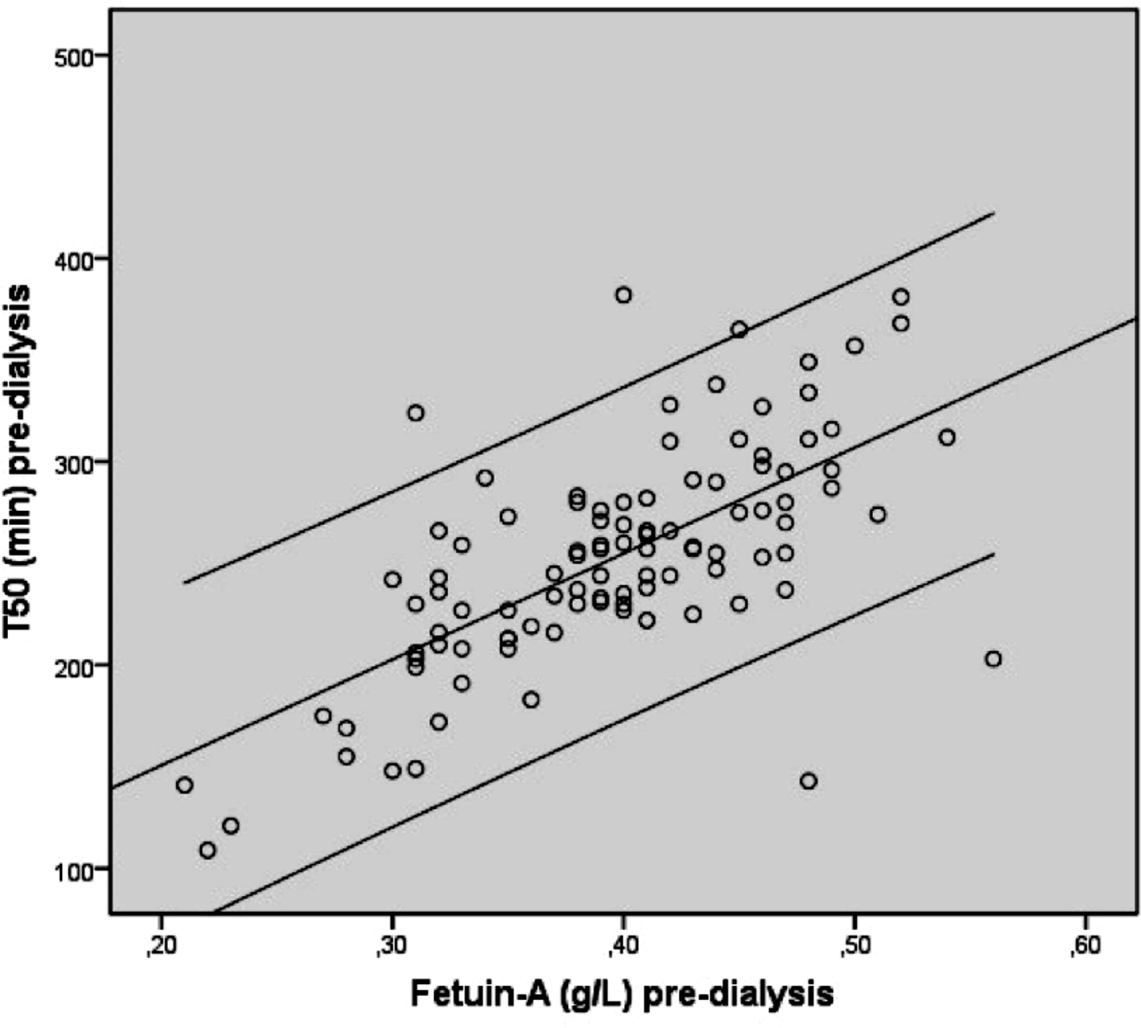
Bivariate correlation of pooled pre-dialysis T_50_ (minutes) and fetuin-A (g/L) analysis in hemodiafiltration patients. Fig 3 (legend): *r* 0.671 P<0.001.

There was no difference in pre- and post-dialysis P levels between the HD and HDF group, nor between the change in P levels in both groups (Table 1, Tables 2 and 3*)*. When the laboratory tests were analysed by bivariate correlation analysis a significant correlation was found between pre-dialysis T_50_ and fetuin-A concentrations in both the HD and HDF group (*r =* 0.731 P<0.001, *r =* 0.671 P<0.001) (Figs 2 and 3). CRP did not significantly correlate with fetuin-A or T_50_ levels (*r =* -0.17 P = 0.2, *r =* -0.12 P = 0.4). Serum magnesium was correlated with pre-dialysis T_50_ in the HD group (*r =* 0.269 P = 0.01) and HDF group (*r =* 0.261 P = 0.01), and with post-dialysis T_50_ in the HD group (*r =* 0.239 P = 0.03) (Tables 7 and 8*)*. Serum fetuin-A values were not influenced by serum magnesium. No significant relations between Ca concentrations and T_50_ were found pre- or post-dialysis in the HD (*r =* -0.067 P = 0.55, *r =* -0.076 P = 0.49) or the HDF group (*r =* -0.089 P = 0.39, *r =* -0.186 P = 0.07). The ΔT_50_ was significantly correlated with the dialysate bicarbonate concentration in the HDF group (*r =* 0.377 P = 0.03), but not in the HD group (*r =* 0.336 P = 0.08). There was also a correlation between the change in pre- and post-dialysis P levels (delta phosphate; ΔP) and the T_50_ in both groups (Tables 7 and 8). However, there was no relation between changes in T_50_, and changes in (corrected) Ca and magnesium in both groups.

**Table 7:** Bivariate correlation pooled analyses of high-flux hemodialysis patients.

	Pre-dialysis		Post-dialysis		Delta	
	*r*	P	*r*	P	*r*	P
T_50_ (min) and phosphate (mmol/L)	**-0.350**	**0.001**	0.108	0.32	**-0.342**	**0.002**
T_50_ (min) and magnesium (mmol/L)	**0.296**	**0.01**	**0.239**	**0.03**	0.127	0.25
T_50_ (min) and calcium (mmol/L)*	-0.067	0.55	-0.076	0.489	0.050	0.65
T_50_ (min) and albumin (g/L)	**0.318**	**0.003**	**0.481**	**<0.001**	0.178	0.11
T_50_ (min) and fetuin-A (g/L)	**0.713**	**<0.001**	**0.776**	**<0.001**	0.183	0.10
T_50_ (min) and bicarbonate (mmol/L)	0.121	0.54				
ΔT_50_ (min) and dialysate bicarbonate (mmol/L)	0.336	0.08				

T_50_: transition time from primary to secondary calciprotein particles.

*Calcium is corrected for serum albumin (g/L).

**Table 8:** Bivariate correlation pooled analyses of high-volume hemodiafiltration patients.

	Pre-dialysis		Post-dialysis		Delta	
	*r*	P	*r*	P	*r*	P
T_50_ (min) and phosphate (mmol/L)	-0.035	0.73	**-0.227**	**0.03**	**-0.396**	**<0.0001**
T_50_ (min) and magnesium (mmol/L)	**0.261**	**0.01**	0.080	0.44	-0.041	0.25
T_50_ (min) and calcium (mmol/L)*	-0.089	0.39	-0.186	0.07	0.011	0.92
T_50_ (min) and albumin (g/L)	**0.416**	**<0.001**	**0.493**	**<0.001**	0.084	0.42
T_50_ (min) and fetuin-A (g/L)	**0.671**	**<0.001**	**0.764**	**<0.001**	0.144	0.16
T_50_ (min) and bicarbonate (mmol/L)	0.238	0.18				
ΔT_50_ (min) and dialysate bicarbonate (mmol/L)	**0.377**	**0.03**				

T_50_: transition time from primary to secondary calciprotein particles.

*Calcium is corrected for serum albumin (g/L).

## Discussion

In this cross-sectional single centre study, we found a significant improvement of calcification propensity of serum, reflected by a higher T_50_, after both HD and HDF without significant differences between both treatment modalities. Both after HD and after HDF, the increase in T_50_ was related to the reduction of serum P levels. Both serum fetuin-A levels and serum magnesium levels were positively related to T_50_. Fetuin-A levels corrected for hemoconcentration decreased slightly but significantly during HDF, but not during HD.

To the best of our knowledge this is the first study to investigate the effect of different dialysis treatment modalities on calcification propensity in dialysis patients. The presence of CPPs in dialysis patients may be a reflection of pathological calcification status in these patients, potentially leading to increased vascular calcification and higher mortality rates. In previous studies by Smith et al. and Pasch et al., respectively, high CPP levels and low T_50_ values were found in CKD patients and associated with increased mortality and increased vascular stiffness [12, 13]. In earlier studies, fetuin-A mineral-complexes (FMCs) (another name for CPPs) were found in rats with renal failure, which was related to increased vascular calcification [16]. Also, increasing levels of FMCs were found in 73 HD patients by Hamano et al. In this study however they did not look into the influence of dialysis on FMC concentration [30].

Fetuin-A was shown in previous studies to be an integral part of CPP formation. On the other hand, serum fetuin-A levels were positively related to T_50_ and thus to the transformation from primary to secondary CPPs [3, 10, 16, 30], confirming its inhibitory effect on calcification propensity. We also confirmed a strong correlation between fetuin-A and T_50_ in both HD and HDF patients. It has been suggested that at low levels, CPP levels might initially be protective against the calcification and inflammatory effects of hydroxyapatite crystals, whereas their pro-inflammatory and pro-calcifying effects may become apparent at high concentrations, such as observed in renal failure [9].

We did not find a difference between HD and HDF in the effect on calcification propensity, as measured by T_50_, in this study. Differences in uremic toxin removal were shown between HD and HDF patients in recent reviews and RCTs between HD and HDF patients, with improved P removal in the HDF group [6, 17, 31]. In the study of Penne et al., lower pre-dialysis P levels were observed with HDF as compared to low flux dialysis [22]. However we did not observe a difference in pre- or post-dialysis P levels in HDF group compared to the HD group, although the convection volumes in this study were comparable to those reported in the literature [31]. This could be due to the fact that high-flux membranes were used in our present study, comparable to studies of others were also no significant differences between HD and HDF were found [24].

Further indication that T_50_ is a modifiable risk factor comes from the finding that changes in serum P were strongly related to calcification propensity in both HD and HDF patients. This is in agreement with earlier studies, where P was shown as the initiator of CPP complex formation and accelerating T_50_ [3, 13]. However, to the best of our knowledge, the relation between electrolyte changes during dialysis and changes in T_50_, in different dialysis modalities, have not been studies yet. Moreover, also serum magnesium levels were positively related to T_50_, which is in agreement with the proposed anti-calcifying effects of this mineral [32]. In contrast to HD, a small effect of HDF on serum fetuin-A levels, corrected for hemoconcentration was observed (P = 0.002), probably due to the fact that the molecular weight is close to the cut-off level of a high flux membrane, this might suggest some removal by the HDF process. However, this small change apparently did not have a negative effect on calcification propensity during HDF, as reflected by T_50_. Interestingly, changes in corrected serum Ca were not related to changes in T_50_. However, in order to shed more light on the potential effects of changes in serum Ca on calcification propensity during dialysis, controlled studies using different dialysate Ca concentrations are needed.

No relation between CRP levels and T_50_ was observed in our study, which is in agreement with published clinical data [12].

Apart from the uncontrolled design, a limitation of the study design is that we did not correlate these factors to outcome so we were not able to investigate long-term effects of dialysis modality on calcification propensity. Moreover, apart from fetuin-A and electrolytes, we did not measure other uremic toxins which might be of relevance in the calcification propensity in dialysis patients, such as FGF-23 and sclerostin [25–27]. In addition, possible effects of dialysate membranes, anticoagulation usage and the impact of different electrolyte and bicarbonate concentrations on CPP-T50, have not been investigated in this current study and should be addressed in future controlled studies. As patients were assigned to HD or HDF for various reasons, selection bias cannot be excluded with certainly. Although the HD and HDF groups appeared well balanced by age, there was a gender difference between both groups.

Summarizing, whereas this study showed a significant and positive effect of HD and HDF on calcification propensity as measured by T_50_, a difference between HD and HDF was not found. Serum P, magnesium, and fetuin-A levels during dialysis were strongly related to calcification propensity. Further studies addressing the potential benefits of dialysis treatment to improve the calcification propensity, e.g. by using higher convection volumes in HDF, or modifying dialysate Ca levels or citrate-containing solutions need to be conducted.

## Supporting Information

S1 Data This is the deidentified datafile used for all the analyses in this manuscript. (XLSX)
