# Correction: High-Flux Hemodialysis and High-Volume Hemodiafiltration Improve Serum Calcification Propensity

**DOI:** 10.1371/journal.pone.0156923

**Published:** 2016-06-01

**Authors:** The PLOS ONE Staff

The following information is missing from the Competing Interests statement: Calciscon AG holds a patent for the T50-Test and markets the T50-Test. The publisher apologizes for this error. The complete, correct Competing Interests statement is: The authors have the following interests. This study was supported by an unrestricted grant from Fresenius MC, Bad Homburg, Germany. Bernard Canaud is employed by Fresenius MC and holds shares in this company. Andreas Pasch is a part-time employee of Calciscon and holds shares in this company. Mauro Dionisi and Matthias Meier are both employed by Calciscon. Calciscon AG holds a patent for the T50-Test and markets the T50-Test. There are no other patents, products in development or marketed products to declare. This does not alter the authors' adherence to PLOS ONE policies on sharing data and materials.

## References

[1] Dekker M, Pasch A, van der Sande F, Konings C, Bachtler M, Dionisi M, et al (2016) High-Flux Hemodialysis and High-Volume Hemodiafiltration Improve Serum Calcification Propensity. PLoS ONE 11(4): e0151508 doi: 10.1371/journal.pone.0151508 27064679 10.1371/journal.pone.0151508 PMC4827813

